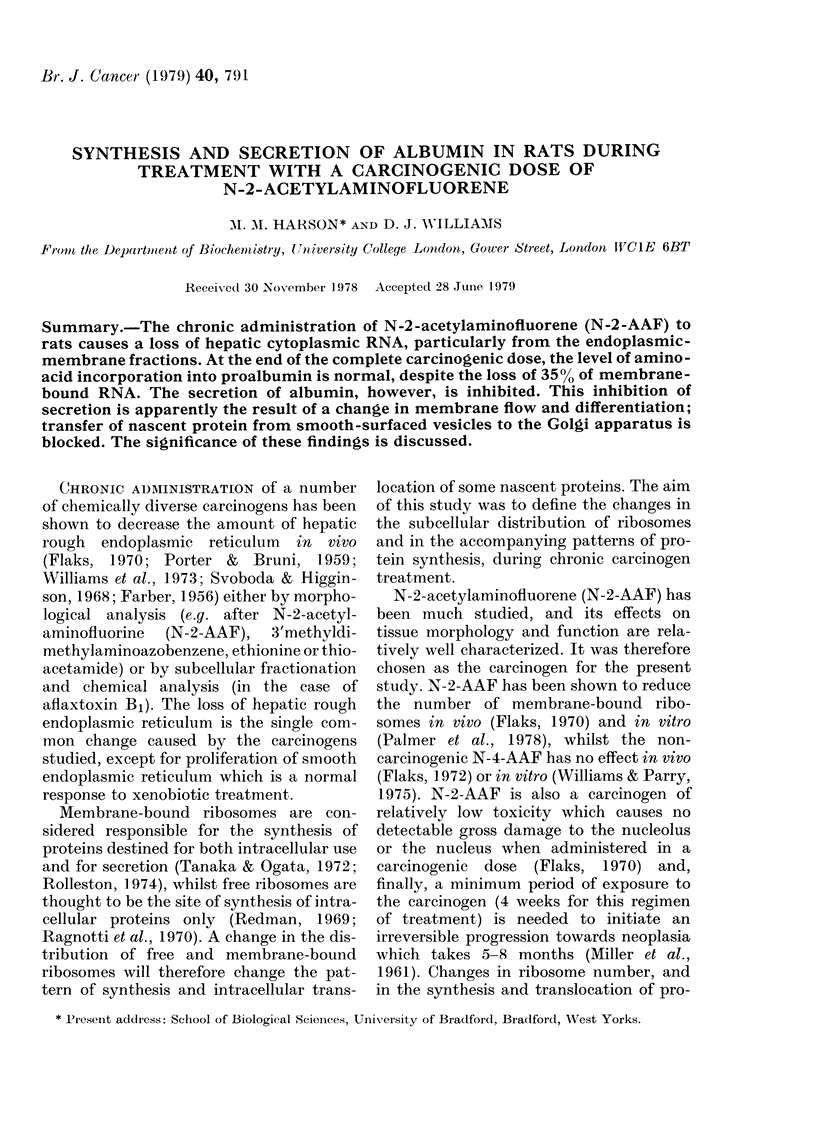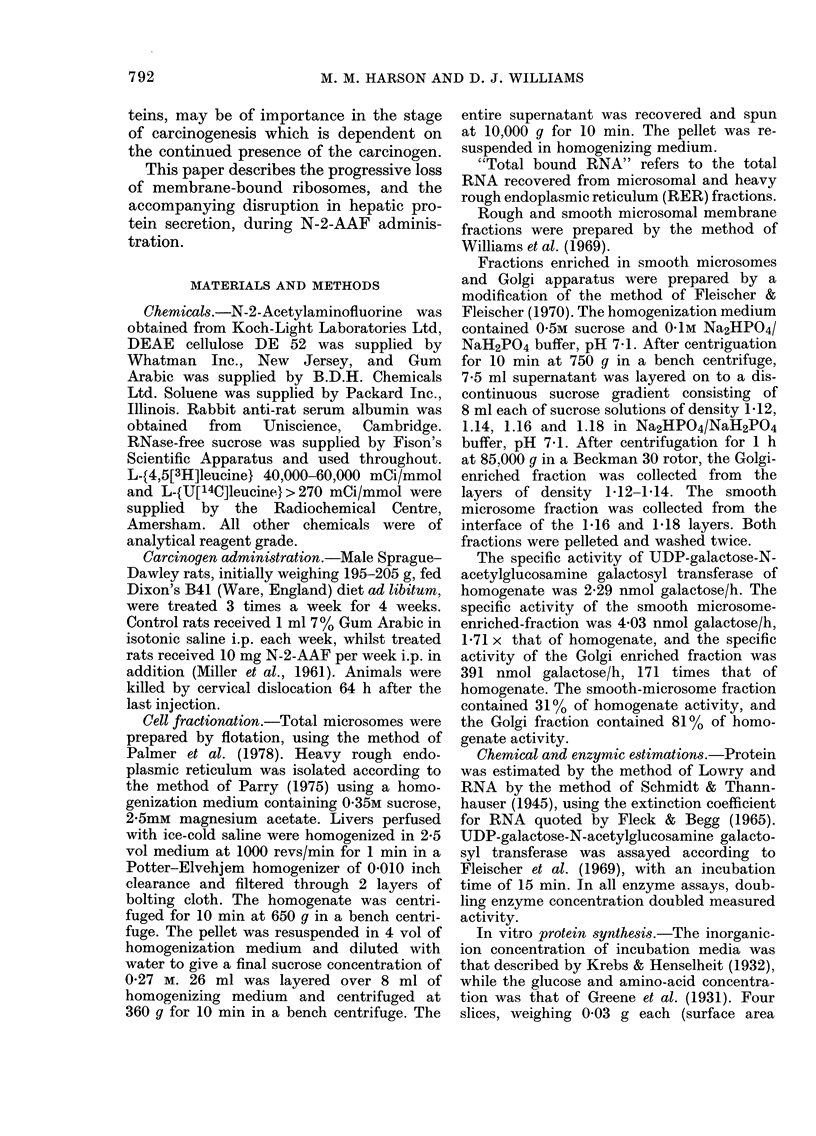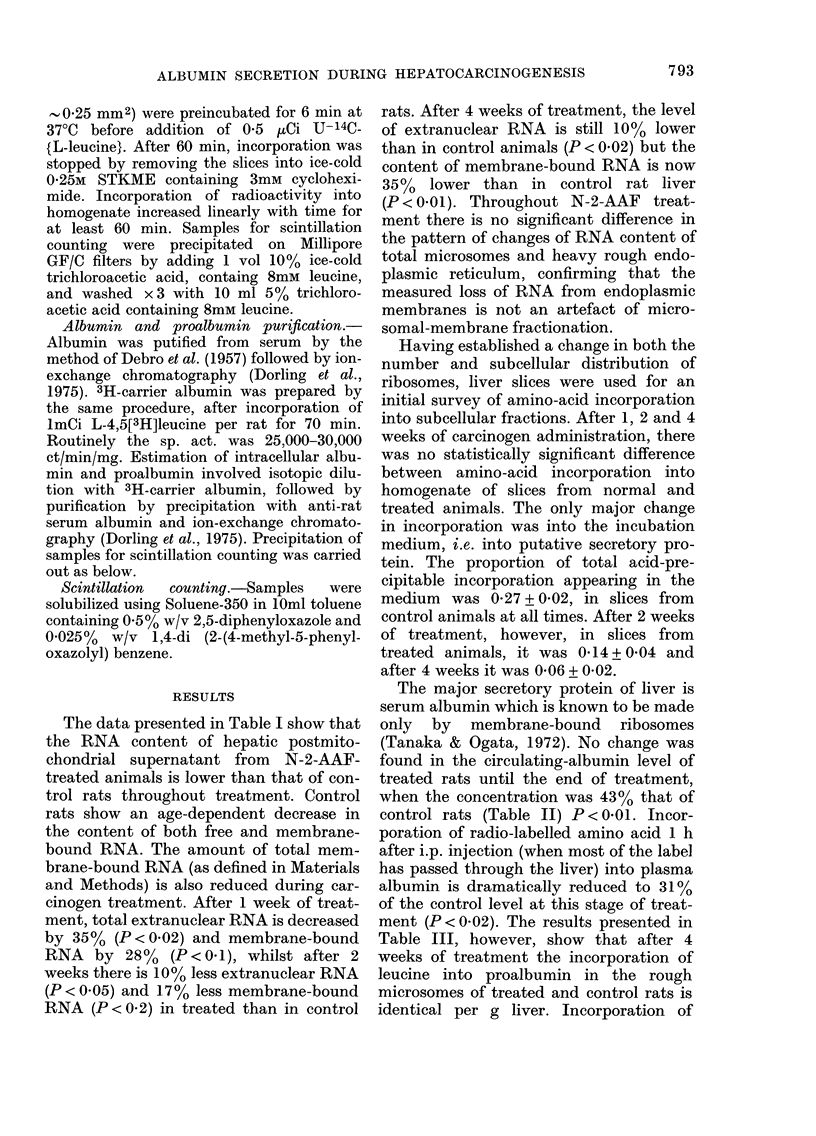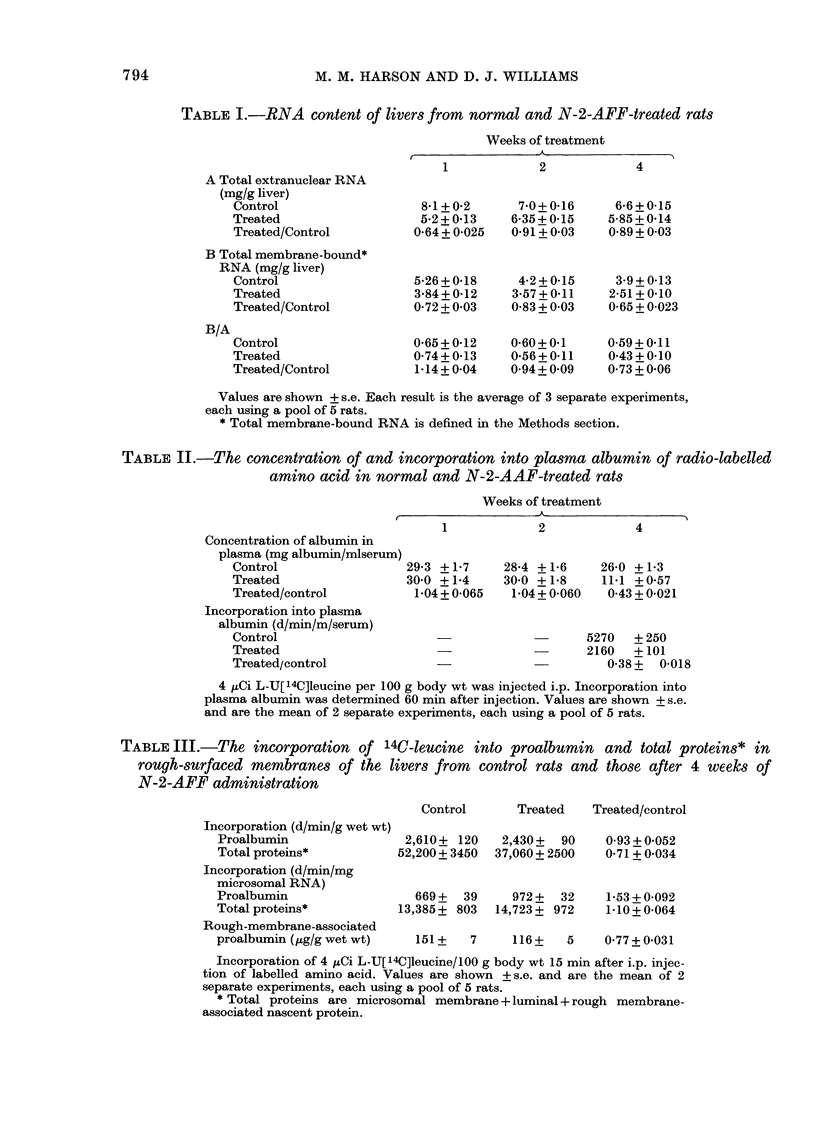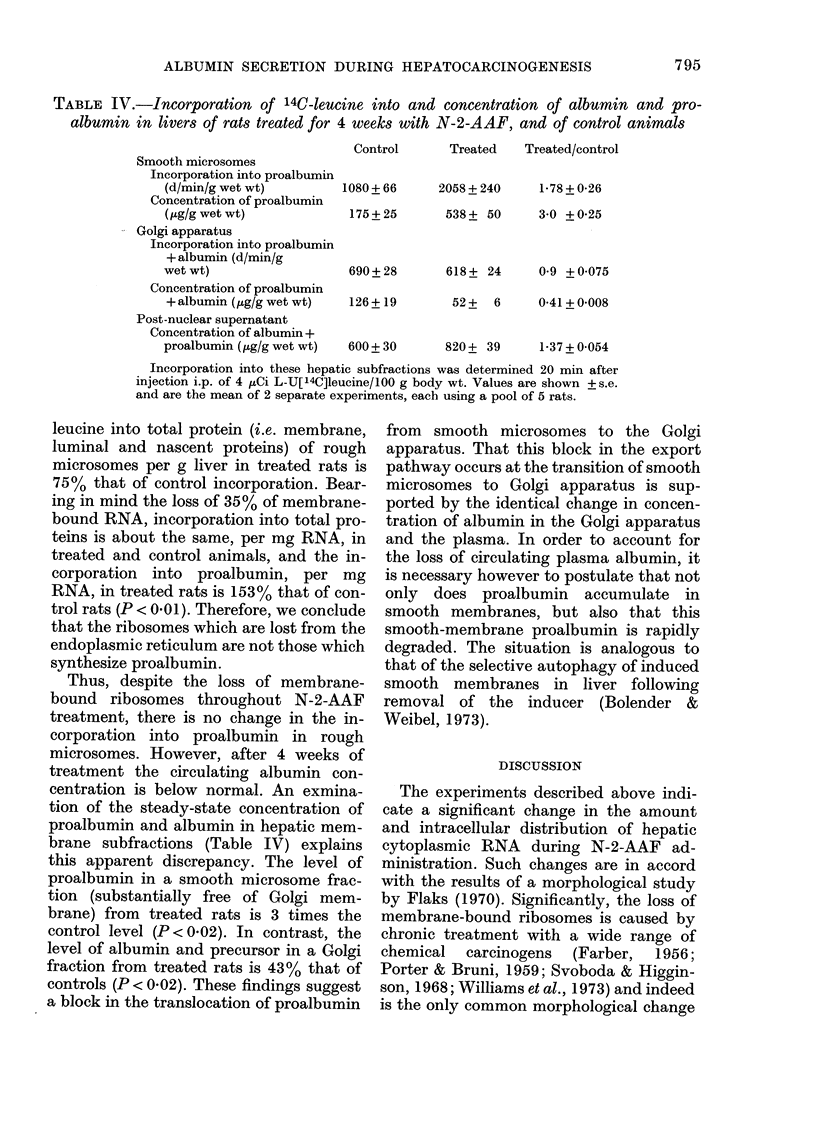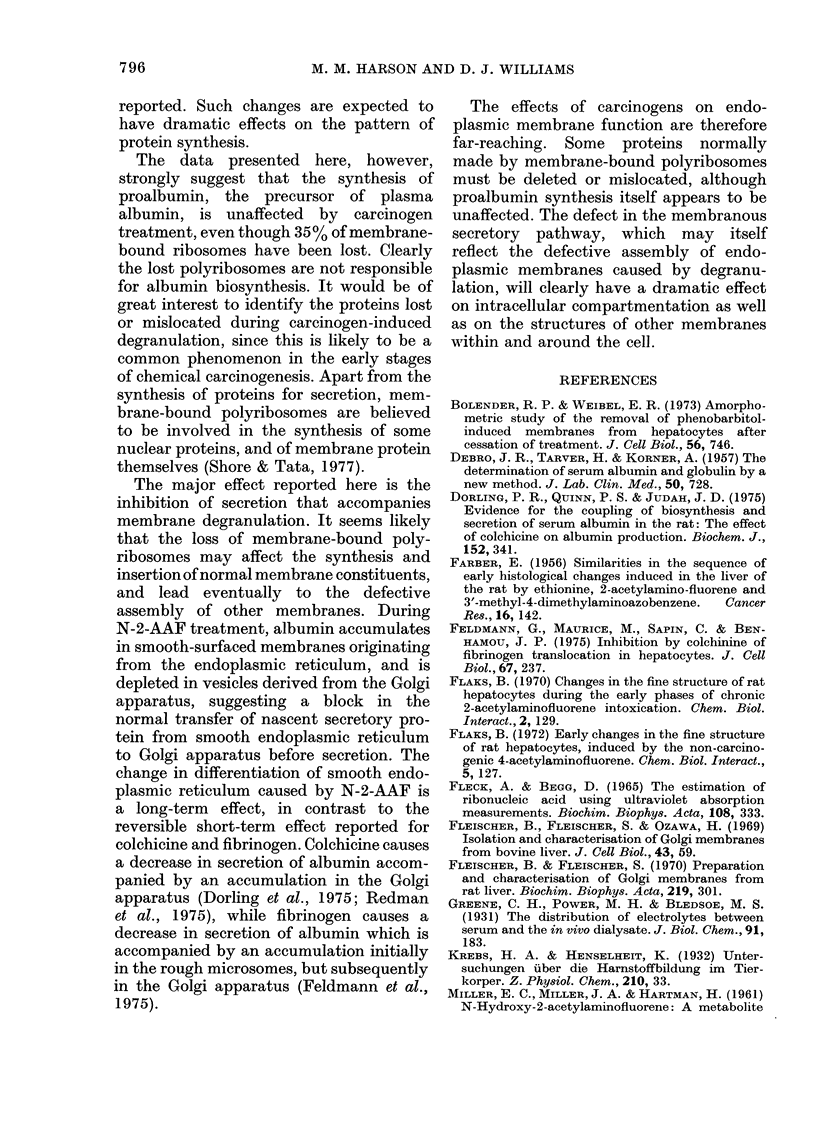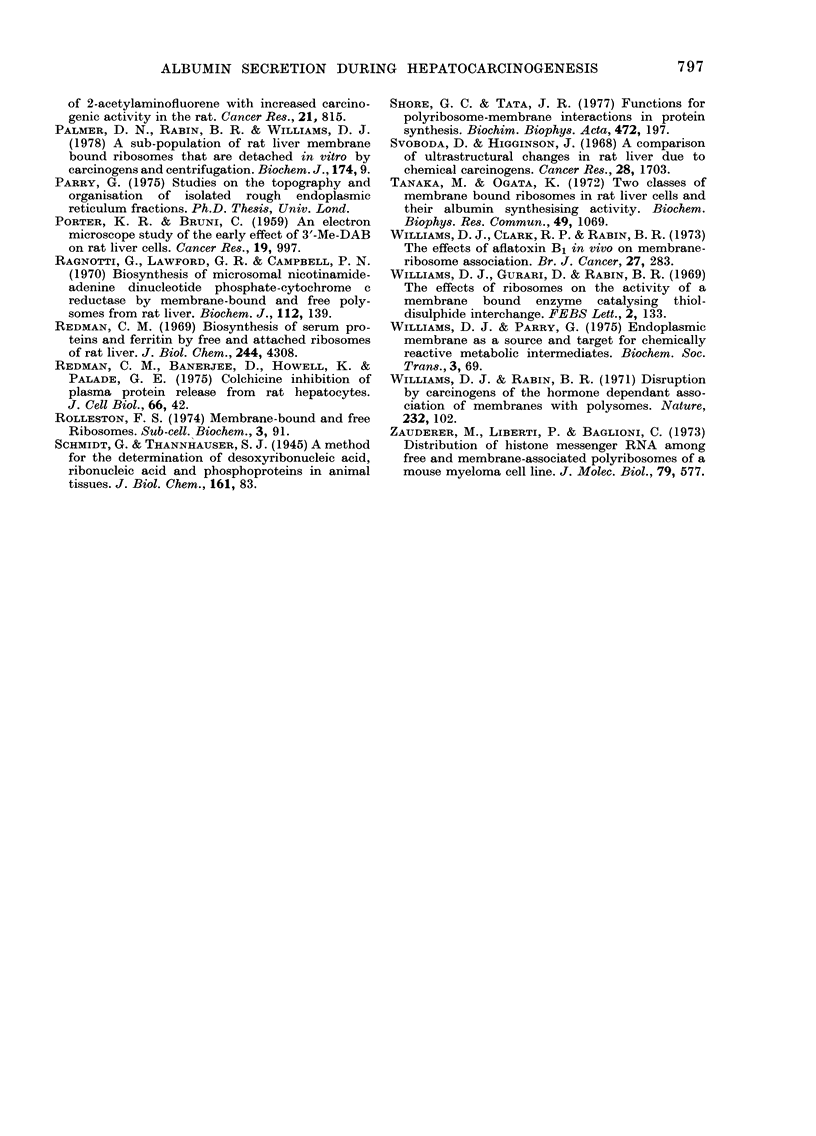# Synthesis and secretion of albumin in rats during treatment with a carcinogenic dose of N-2-acetylaminofluorene.

**DOI:** 10.1038/bjc.1979.262

**Published:** 1979-11

**Authors:** M. M. Harson, D. J. Williams

## Abstract

The chronic administration of N-2-acetylaminofluorene (N-2-AAF) to rats causes a loss of hepatic cytoplasmic RNA, particularly from the endoplasmic-membrane fractions. At the end of the complete carcinogenic dose, the level of amino-acid incorporation into proalbumin is normal, despite the loss of 35% of membrane-bound RNA. The secretion of albumin, however, is inhibited. This inhibition of secretion is apparently the result of a change in membrane flow and differentiation; transfer of nascent protein from smooth-surfaced vesicles to the Golgi apparatus is blocked. The significance of these findings is discussed.


*Br. J. Cancer* (1979) **40**, 791

### SYNTHESIS AND SECRETION OF ALBUMIN IN RATS DURING TREATMENT WITH A CARCINOGENIC DOSE OF N-2-ACETYLAMINOFLUORENE

M. M. HARSON* AND D. J. WILLIAMS

*From the Department of Biochemistry, University College London, Gower Street, London WC1E 6BT*

Received 30 November 1978 Accepted 28 June 1979

**Summary.—The chronic administration of N-2-acetylaminofluorene (N-2-AAF) to rats causes a loss of hepatic cytoplasmic RNA, particularly from the endoplasmic-membrane fractions. At the end of the complete carcinogenic dose, the level of amino-acid incorporation into proalbumin is normal, despite the loss of 35% of membrane-bound RNA. The secretion of albumin, however, is inhibited. This inhibition of secretion is apparently the result of a change in membrane flow and differentiation; transfer of nascent protein from smooth-surfaced vesicles to the Golgi apparatus is blocked. The significance of these findings is discussed.**

CHRONIC ADMINISTRATION of a number of chemically diverse carcinogens has been shown to decrease the amount of hepatic rough endoplasmic reticulum *in vivo* (Flaks, 1970; Porter & Bruni, 1959; Williams *et al.*, 1973; Svoboda & Higginson, 1968; Farber, 1956) either by morphological analysis (*e.g.* after N-2-acetylaminofluorine (N-2-AAF), 3′methyldimethylaminoazobenzene, ethionine or thioacetamide) or by subcellular fractionation and chemical analysis (in the case of aflaxtoxin $B_1$). The loss of hepatic rough endoplasmic reticulum is the single common change caused by the carcinogens studied, except for proliferation of smooth endoplasmic reticulum which is a normal response to xenobiotic treatment.

Membrane-bound ribosomes are considered responsible for the synthesis of proteins destined for both intracellular use and for secretion (Tanaka & Ogata, 1972; Rolleston, 1974), whilst free ribosomes are thought to be the site of synthesis of intracellular proteins only (Redman, 1969; Ragnotti *et al.*, 1970). A change in the distribution of free and membrane-bound ribosomes will therefore change the pattern of synthesis and intracellular translocation of some nascent proteins. The aim of this study was to define the changes in the subcellular distribution of ribosomes and in the accompanying patterns of protein synthesis, during chronic carcinogen treatment.

N-2-acetylaminofluorene (N-2-AAF) has been much studied, and its effects on tissue morphology and function are relatively well characterized. It was therefore chosen as the carcinogen for the present study. N-2-AAF has been shown to reduce the number of membrane-bound ribosomes *in vivo* (Flaks, 1970) and *in vitro* (Palmer *et al.*, 1978), whilst the non-carcinogenic N-4-AAF has no effect *in vivo* (Flaks, 1972) or *in vitro* (Williams & Parry, 1975). N-2-AAF is also a carcinogen of relatively low toxicity which causes no detectable gross damage to the nucleolus or the nucleus when administered in a carcinogenic dose (Flaks, 1970) and, finally, a minimum period of exposure to the carcinogen (4 weeks for this regimen of treatment) is needed to initiate an irreversible progression towards neoplasia which takes 5–8 months (Miller *et al.*, 1961). Changes in ribosome number, and in the synthesis and translocation of pro-

* Present address: School of Biological Sciences, University of Bradford, Bradford, West Yorks.

teins, may be of importance in the stage of carcinogenesis which is dependent on the continued presence of the carcinogen.

This paper describes the progressive loss of membrane-bound ribosomes, and the accompanying disruption in hepatic protein secretion, during N-2-AAF administration.

### MATERIALS AND METHODS

*Chemicals.*—N-2-Acetylaminofluorine was obtained from Koch-Light Laboratories Ltd, DEAE cellulose DE 52 was supplied by Whatman Inc., New Jersey, and Gum Arabic was supplied by B.D.H. Chemicals Ltd. Soluene was supplied by Packard Inc., Illinois. Rabbit anti-rat serum albumin was obtained from Uniscience, Cambridge. RNase-free sucrose was supplied by Fison's Scientific Apparatus and used throughout. L-{4,5[$^3$H]leucine} 40,000–60,000 mCi/mmol and L-{U[$^{14}$C]leucine} > 270 mCi/mmol were supplied by the Radiochemical Centre, Amersham. All other chemicals were of analytical reagent grade.

*Carcinogen administration.*—Male Sprague–Dawley rats, initially weighing 195–205 g, fed Dixon's B41 (Ware, England) diet *ad libitum*, were treated 3 times a week for 4 weeks. Control rats received 1 ml 7% Gum Arabic in isotonic saline i.p. each week, whilst treated rats received 10 mg N-2-AAF per week i.p. in addition (Miller *et al.*, 1961). Animals were killed by cervical dislocation 64 h after the last injection.

*Cell fractionation.*—Total microsomes were prepared by flotation, using the method of Palmer *et al.* (1978). Heavy rough endoplasmic reticulum was isolated according to the method of Parry (1975) using a homogenization medium containing 0·35M sucrose, 2·5mM magnesium acetate. Livers perfused with ice-cold saline were homogenized in 2·5 vol medium at 1000 revs/min for 1 min in a Potter–Elvehjem homogenizer of 0·010 inch clearance and filtered through 2 layers of bolting cloth. The homogenate was centrifuged for 10 min at 650 *g* in a bench centrifuge. The pellet was resuspended in 4 vol of homogenization medium and diluted with water to give a final sucrose concentration of 0·27 M. 26 ml was layered over 8 ml of homogenizing medium and centrifuged at 360 *g* for 10 min in a bench centrifuge. The entire supernatant was recovered and spun at 10,000 *g* for 10 min. The pellet was resuspended in homogenizing medium.

"Total bound RNA" refers to the total RNA recovered from microsomal and heavy rough endoplasmic reticulum (RER) fractions.

Rough and smooth microsomal membrane fractions were prepared by the method of Williams *et al.* (1969).

Fractions enriched in smooth microsomes and Golgi apparatus were prepared by a modification of the method of Fleischer & Fleischer (1970). The homogenization medium contained 0·5M sucrose and 0·1M $Na_2HPO_4$/$NaH_2PO_4$ buffer, pH 7·1. After centriguation for 10 min at 750 *g* in a bench centrifuge, 7·5 ml supernatant was layered on to a discontinuous sucrose gradient consisting of 8 ml each of sucrose solutions of density 1·12, 1.14, 1.16 and 1.18 in $Na_2HPO_4$/$NaH_2PO_4$ buffer, pH 7·1. After centrifugation for 1 h at 85,000 *g* in a Beckman 30 rotor, the Golgi-enriched fraction was collected from the layers of density 1·12–1·14. The smooth microsome fraction was collected from the interface of the 1·16 and 1·18 layers. Both fractions were pelleted and washed twice.

The specific activity of UDP-galactose-N-acetylglucosamine galactosyl transferase of homogenate was 2·29 nmol galactose/h. The specific activity of the smooth microsome-enriched-fraction was 4·03 nmol galactose/h, 1·71 × that of homogenate, and the specific activity of the Golgi enriched fraction was 391 nmol galactose/h, 171 times that of homogenate. The smooth-microsome fraction contained 31% of homogenate activity, and the Golgi fraction contained 81% of homogenate activity.

*Chemical and enzymic estimations.*—Protein was estimated by the method of Lowry and RNA by the method of Schmidt & Thannhauser (1945), using the extinction coefficient for RNA quoted by Fleck & Begg (1965). UDP-galactose-N-acetylglucosamine galactosyl transferase was assayed according to Fleischer *et al.* (1969), with an incubation time of 15 min. In all enzyme assays, doubling enzyme concentration doubled measured activity.

In vitro *protein synthesis.*—The inorganic-ion concentration of incubation media was that described by Krebs & Henselheit (1932), while the glucose and amino-acid concentration was that of Greene *et al.* (1931). Four slices, weighing 0·03 g each (surface area

$\sim$0·25 mm$^2$) were preincubated for 6 min at 37°C before addition of 0·5 $\mu$Ci U-$^{14}$C-{L-leucine}. After 60 min, incorporation was stopped by removing the slices into ice-cold 0·25M STKME containing 3mM cycloheximide. Incorporation of radioactivity into homogenate increased linearly with time for at least 60 min. Samples for scintillation counting were precipitated on Millipore GF/C filters by adding 1 vol 10% ice-cold trichloroacetic acid, containg 8mM leucine, and washed $\times$3 with 10 ml 5% trichloroacetic acid containing 8mM leucine.

*Albumin and proalbumin purification.*—Albumin was putified from serum by the method of Debro *et al.* (1957) followed by ion-exchange chromatography (Dorling *et al.*, 1975). $^3$H-carrier albumin was prepared by the same procedure, after incorporation of 1mCi L-4,5[$^3$H]leucine per rat for 70 min. Routinely the sp. act. was 25,000–30,000 ct/min/mg. Estimation of intracellular albumin and proalbumin involved isotopic dilution with $^3$H-carrier albumin, followed by purification by precipitation with anti-rat serum albumin and ion-exchange chromatography (Dorling *et al.*, 1975). Precipitation of samples for scintillation counting was carried out as below.

*Scintillation counting.*—Samples were solubilized using Soluene-350 in 10ml toluene containing 0·5% w/v 2,5-diphenyloxazole and 0·025% w/v 1,4-di (2-(4-methyl-5-phenyloxazolyl) benzene.

### RESULTS

The data presented in Table I show that the RNA content of hepatic postmitochondrial supernatant from N-2-AAF-treated animals is lower than that of control rats throughout treatment. Control rats show an age-dependent decrease in the content of both free and membrane-bound RNA. The amount of total membrane-bound RNA (as defined in Materials and Methods) is also reduced during carcinogen treatment. After 1 week of treatment, total extranuclear RNA is decreased by 35% ($P < 0·02$) and membrane-bound RNA by 28% ($P < 0·1$), whilst after 2 weeks there is 10% less extranuclear RNA ($P < 0·05$) and 17% less membrane-bound RNA ($P < 0·2$) in treated than in control rats. After 4 weeks of treatment, the level of extranuclear RNA is still 10% lower than in control animals ($P < 0·02$) but the content of membrane-bound RNA is now 35% lower than in control rat liver ($P < 0·01$). Throughout N-2-AAF treatment there is no significant difference in the pattern of changes of RNA content of total microsomes and heavy rough endoplasmic reticulum, confirming that the measured loss of RNA from endoplasmic membranes is not an artefact of microsomal-membrane fractionation.

Having established a change in both the number and subcellular distribution of ribosomes, liver slices were used for an initial survey of amino-acid incorporation into subcellular fractions. After 1, 2 and 4 weeks of carcinogen administration, there was no statistically significant difference between amino-acid incorporation into homogenate of slices from normal and treated animals. The only major change in incorporation was into the incubation medium, *i.e.* into putative secretory protein. The proportion of total acid-precipitable incorporation appearing in the medium was $0·27 \pm 0·02$, in slices from control animals at all times. After 2 weeks of treatment, however, in slices from treated animals, it was $0·14 \pm 0·04$ and after 4 weeks it was $0·06 \pm 0·02$.

The major secretory protein of liver is serum albumin which is known to be made only by membrane-bound ribosomes (Tanaka & Ogata, 1972). No change was found in the circulating-albumin level of treated rats until the end of treatment, when the concentration was 43% that of control rats (Table II) $P < 0·01$. Incorporation of radio-labelled amino acid 1 h after i.p. injection (when most of the label has passed through the liver) into plasma albumin is dramatically reduced to 31% of the control level at this stage of treatment ($P < 0·02$). The results presented in Table III, however, show that after 4 weeks of treatment the incorporation of leucine into proalbumin in the rough microsomes of treated and control rats is identical per g liver. Incorporation of

TABLE I.—*RNA content of livers from normal and N-2-AFF-treated rats*

| | Weeks of treatment | | |
|---|---|---|---|
| | 1 | 2 | 4 |
| A Total extranuclear RNA (mg/g liver) | | | |
| Control | 8·1 ± 0·2 | 7·0 ± 0·16 | 6·6 ± 0·15 |
| Treated | 5·2 ± 0·13 | 6·35 ± 0·15 | 5·85 ± 0·14 |
| Treated/Control | 0·64 ± 0·025 | 0·91 ± 0·03 | 0·89 ± 0·03 |
| B Total membrane-bound* RNA (mg/g liver) | | | |
| Control | 5·26 ± 0·18 | 4·2 ± 0·15 | 3·9 ± 0·13 |
| Treated | 3·84 ± 0·12 | 3·57 ± 0·11 | 2·51 ± 0·10 |
| Treated/Control | 0·72 ± 0·03 | 0·83 ± 0·03 | 0·65 ± 0·023 |
| B/A | | | |
| Control | 0·65 ± 0·12 | 0·60 ± 0·1 | 0·59 ± 0·11 |
| Treated | 0·74 ± 0·13 | 0·56 ± 0·11 | 0·43 ± 0·10 |
| Treated/Control | 1·14 ± 0·04 | 0·94 ± 0·09 | 0·73 ± 0·06 |

Values are shown ± s.e. Each result is the average of 3 separate experiments, each using a pool of 5 rats.
* Total membrane-bound RNA is defined in the Methods section.

TABLE II.—*The concentration of and incorporation into plasma albumin of radio-labelled amino acid in normal and N-2-AAF-treated rats*

| | Weeks of treatment | | |
|---|---|---|---|
| | 1 | 2 | 4 |
| Concentration of albumin in plasma (mg albumin/mlserum) | | | |
| Control | 29·3 ± 1·7 | 28·4 ± 1·6 | 26·0 ± 1·3 |
| Treated | 30·0 ± 1·4 | 30·0 ± 1·8 | 11·1 ± 0·57 |
| Treated/control | 1·04 ± 0·065 | 1·04 ± 0·060 | 0·43 ± 0·021 |
| Incorporation into plasma albumin (d/min/m/serum) | | | |
| Control | — | — | 5270 ± 250 |
| Treated | — | — | 2160 ± 101 |
| Treated/control | — | — | 0·38 ± 0·018 |

4 $\mu$Ci L-U[$^{14}$C]leucine per 100 g body wt was injected i.p. Incorporation into plasma albumin was determined 60 min after injection. Values are shown ± s.e. and are the mean of 2 separate experiments, each using a pool of 5 rats.

TABLE III.—*The incorporation of* $^{14}$*C-leucine into proalbumin and total proteins* in rough-surfaced membranes of the livers from control rats and those after* 4 *weeks of N-2-AFF administration*

| | Control | Treated | Treated/control |
|---|---|---|---|
| Incorporation (d/min/g wet wt) | | | |
| Proalbumin | 2,610 ± 120 | 2,430 ± 90 | 0·93 ± 0·052 |
| Total proteins* | 52,200 ± 3450 | 37,060 ± 2500 | 0·71 ± 0·034 |
| Incorporation (d/min/mg microsomal RNA) | | | |
| Proalbumin | 669 ± 39 | 972 ± 32 | 1·53 ± 0·092 |
| Total proteins* | 13,385 ± 803 | 14,723 ± 972 | 1·10 ± 0·064 |
| Rough-membrane-associated proalbumin ($\mu$g/g wet wt) | 151 ± 7 | 116 ± 5 | 0·77 ± 0·031 |

Incorporation of 4 $\mu$Ci L-U[$^{14}$C]leucine/100 g body wt 15 min after i.p. injection of labelled amino acid. Values are shown ± s.e. and are the mean of 2 separate experiments, each using a pool of 5 rats.
* Total proteins are microsomal membrane + luminal + rough membrane-associated nascent protein.

TABLE IV.—*Incorporation of $^{14}C$-leucine into and concentration of albumin and proalbumin in livers of rats treated for 4 weeks with N-2-AAF, and of control animals*

| | Control | Treated | Treated/control |
|---|---|---|---|
| Smooth microsomes | | | |
| Incorporation into proalbumin (d/min/g wet wt) | 1080 ± 66 | 2058 ± 240 | 1·78 ± 0·26 |
| Concentration of proalbumin (μg/g wet wt) | 175 ± 25 | 538 ± 50 | 3·0 ± 0·25 |
| Golgi apparatus | | | |
| Incorporation into proalbumin + albumin (d/min/g wet wt) | 690 ± 28 | 618 ± 24 | 0·9 ± 0·075 |
| Concentration of proalbumin + albumin (μg/g wet wt) | 126 ± 19 | 52 ± 6 | 0·41 ± 0·008 |
| Post-nuclear supernatant | | | |
| Concentration of albumin + proalbumin (μg/g wet wt) | 600 ± 30 | 820 ± 39 | 1·37 ± 0·054 |

Incorporation into these hepatic subfractions was determined 20 min after injection i.p. of 4 μCi L-U[$^{14}$C]leucine/100 g body wt. Values are shown ±s.e. and are the mean of 2 separate experiments, each using a pool of 5 rats.

leucine into total protein (*i.e.* membrane, luminal and nascent proteins) of rough microsomes per g liver in treated rats is 75% that of control incorporation. Bearing in mind the loss of 35% of membrane-bound RNA, incorporation into total proteins is about the same, per mg RNA, in treated and control animals, and the incorporation into proalbumin, per mg RNA, in treated rats is 153% that of control rats ($P < 0{\cdot}01$). Therefore, we conclude that the ribosomes which are lost from the endoplasmic reticulum are not those which synthesize proalbumin.

Thus, despite the loss of membrane-bound ribosomes throughout N-2-AAF treatment, there is no change in the incorporation into proalbumin in rough microsomes. However, after 4 weeks of treatment the circulating albumin concentration is below normal. An exmination of the steady-state concentration of proalbumin and albumin in hepatic membrane subfractions (Table IV) explains this apparent discrepancy. The level of proalbumin in a smooth microsome fraction (substantially free of Golgi membrane) from treated rats is 3 times the control level ($P < 0{\cdot}02$). In contrast, the level of albumin and precursor in a Golgi fraction from treated rats is 43% that of controls ($P < 0{\cdot}02$). These findings suggest a block in the translocation of proalbumin from smooth microsomes to the Golgi apparatus. That this block in the export pathway occurs at the transition of smooth microsomes to Golgi apparatus is supported by the identical change in concentration of albumin in the Golgi apparatus and the plasma. In order to account for the loss of circulating plasma albumin, it is necessary however to postulate that not only does proalbumin accumulate in smooth membranes, but also that this smooth-membrane proalbumin is rapidly degraded. The situation is analogous to that of the selective autophagy of induced smooth membranes in liver following removal of the inducer (Bolender & Weibel, 1973).

### DISCUSSION

The experiments described above indicate a significant change in the amount and intracellular distribution of hepatic cytoplasmic RNA during N-2-AAF administration. Such changes are in accord with the results of a morphological study by Flaks (1970). Significantly, the loss of membrane-bound ribosomes is caused by chronic treatment with a wide range of chemical carcinogens (Farber, 1956; Porter & Bruni, 1959; Svoboda & Higginson, 1968; Williams *et al.*, 1973) and indeed is the only common morphological change reported. Such changes are expected to have dramatic effects on the pattern of protein synthesis.

The data presented here, however, strongly suggest that the synthesis of proalbumin, the precursor of plasma albumin, is unaffected by carcinogen treatment, even though 35% of membrane-bound ribosomes have been lost. Clearly the lost polyribosomes are not responsible for albumin biosynthesis. It would be of great interest to identify the proteins lost or mislocated during carcinogen-induced degranulation, since this is likely to be a common phenomenon in the early stages of chemical carcinogenesis. Apart from the synthesis of proteins for secretion, membrane-bound polyribosomes are believed to be involved in the synthesis of some nuclear proteins, and of membrane protein themselves (Shore & Tata, 1977).

The major effect reported here is the inhibition of secretion that accompanies membrane degranulation. It seems likely that the loss of membrane-bound polyribosomes may affect the synthesis and insertion of normal membrane constituents, and lead eventually to the defective assembly of other membranes. During N-2-AAF treatment, albumin accumulates in smooth-surfaced membranes originating from the endoplasmic reticulum, and is depleted in vesicles derived from the Golgi apparatus, suggesting a block in the normal transfer of nascent secretory protein from smooth endoplasmic reticulum to Golgi apparatus before secretion. The change in differentiation of smooth endoplasmic reticulum caused by N-2-AAF is a long-term effect, in contrast to the reversible short-term effect reported for colchicine and fibrinogen. Colchicine causes a decrease in secretion of albumin accompanied by an accumulation in the Golgi apparatus (Dorling *et al.*, 1975; Redman *et al.*, 1975), while fibrinogen causes a decrease in secretion of albumin which is accompanied by an accumulation initially in the rough microsomes, but subsequently in the Golgi apparatus (Feldmann *et al.*, 1975).

The effects of carcinogens on endoplasmic membrane function are therefore far-reaching. Some proteins normally made by membrane-bound polyribosomes must be deleted or mislocated, although proalbumin synthesis itself appears to be unaffected. The defect in the membranous secretory pathway, which may itself reflect the defective assembly of endoplasmic membranes caused by degranulation, will clearly have a dramatic effect on intracellular compartmentation as well as on the structures of other membranes within and around the cell.

### REFERENCES

Bolender, R. P. & Weibel, E. R. (1973) Amorphometric study of the removal of phenobarbitol-induced membranes from hepatocytes after cessation of treatment. *J. Cell Biol.*, **56**, 746.

Debro, J. R., Tarver, H. & Korner, A. (1957) The determination of serum albumin and globulin by a new method. *J. Lab. Clin. Med.*, **50**, 728.

Dorling, P. R., Quinn, P. S. & Judah, J. D. (1975) Evidence for the coupling of biosynthesis and secretion of serum albumin in the rat: The effect of colchicine on albumin production. *Biochem. J.*, **152**, 341.

Farber, E. (1956) Similarities in the sequence of early histological changes induced in the liver of the rat by ethionine, 2-acetylamino-fluorene and 3′-methyl-4-dimethylaminoazobenzene. *Cancer Res.*, **16**, 142.

Feldmann, G., Maurice, M., Sapin, C. & Benhamou, J. P. (1975) Inhibition by colchinine of fibrinogen translocation in hepatocytes. *J. Cell Biol.*, **67**, 237.

Flaks, B. (1970) Changes in the fine structure of rat hepatocytes during the early phases of chronic 2-acetylaminofluorene intoxication. *Chem. Biol. Interact.*, **2**, 129.

Flaks, B. (1972) Early changes in the fine structure of rat hepatocytes, induced by the non-carcinogenic 4-acetylaminofluorene. *Chem. Biol. Interact.*, **5**, 127.

Fleck, A. & Begg, D. (1965) The estimation of ribonucleic acid using ultraviolet absorption measurements. *Biochim. Biophys. Acta*, **108**, 333.

Fleischer, B., Fleischer, S. & Ozawa, H. (1969) Isolation and characterisation of Golgi membranes from bovine liver. *J. Cell Biol.*, **43**, 59.

Fleischer, B. & Fleischer, S. (1970) Preparation and characterisation of Golgi membranes from rat liver. *Biochim. Biophys. Acta*, **219**, 301.

Greene, C. H., Power, M. H. & Bledsoe, M. S. (1931) The distribution of electrolytes between serum and the *in vivo* dialysate. *J. Biol. Chem.*, **91**, 183.

Krebs, H. A. & Henselheit, K. (1932) Untersuchungen über die Harnstoffbildung im Tierkorper. *Z. Physiol. Chem.*, **210**, 33.

Miller, E. C., Miller, J. A. & Hartman, H. (1961) N-Hydroxy-2-acetylaminofluorene: A metabolite of 2-acetylaminofluorene with increased carcinogenic activity in the rat. *Cancer Res.*, **21**, 815.

PALMER, D. N., RABIN, B. R. & WILLIAMS, D. J. (1978) A sub-population of rat liver membrane bound ribosomes that are detached *in vitro* by carcinogens and centrifugation. *Biochem. J.*, **174**, 9.

PARRY, G. (1975) Studies on the topography and organisation of isolated rough endoplasmic reticulum fractions. *Ph.D. Thesis, Univ. Lond.*

PORTER, K. R. & BRUNI, C. (1959) An electron microscope study of the early effect of 3′-Me-DAB on rat liver cells. *Cancer Res.*, **19**, 997.

RAGNOTTI, G., LAWFORD, G. R. & CAMPBELL, P. N. (1970) Biosynthesis of microsomal nicotinamide-adenine dinucleotide phosphate-cytochrome c reductase by membrane-bound and free polysomes from rat liver. *Biochem. J.*, **112**, 139.

REDMAN, C. M. (1969) Biosynthesis of serum proteins and ferritin by free and attached ribosomes of rat liver. *J. Biol. Chem.*, **244**, 4308.

REDMAN, C. M., BANERJEE, D., HOWELL, K. & PALADE, G. E. (1975) Colchicine inhibition of plasma protein release from rat hepatocytes. *J. Cell Biol.*, **66**, 42.

ROLLESTON, F. S. (1974) Membrane-bound and free Ribosomes. *Sub-cell. Biochem.*, **3**, 91.

SCHMIDT, G. & THANNHAUSER, S. J. (1945) A method for the determination of desoxyribonucleic acid, ribonucleic acid and phosphoproteins in animal tissues. *J. Biol. Chem.*, **161**, 83.

SHORE, G. C. & TATA, J. R. (1977) Functions for polyribosome-membrane interactions in protein synthesis. *Biochim. Biophys. Acta*, **472**, 197.

SVOBODA, D. & HIGGINSON, J. (1968) A comparison of ultrastructural changes in rat liver due to chemical carcinogens. *Cancer Res.*, **28**, 1703.

TANAKA, M. & OGATA, K. (1972) Two classes of membrane bound ribosomes in rat liver cells and their albumin synthesising activity. *Biochem. Biophys. Res. Commun.*, **49**, 1069.

WILLIAMS, D. J., CLARK, R. P. & RABIN, B. R. (1973) The effects of aflatoxin $B_1$ *in vivo* on membrane-ribosome association. *Br. J. Cancer*, **27**, 283.

WILLIAMS, D. J., GURARI, D. & RABIN, B. R. (1969) The effects of ribosomes on the activity of a membrane bound enzyme catalysing thiol-disulphide interchange. *FEBS Lett.*, **2**, 133.

WILLIAMS, D. J. & PARRY, G. (1975) Endoplasmic membrane as a source and target for chemically reactive metabolic intermediates. *Biochem. Soc. Trans.*, **3**, 69.

WILLIAMS, D. J. & RABIN, B. R. (1971) Disruption by carcinogens of the hormone dependant association of membranes with polysomes. *Nature*, **232**, 102.

ZAUDERER, M., LIBERTI, P. & BAGLIONI, C. (1973) Distribution of histone messenger RNA among free and membrane-associated polyribosomes of a mouse myeloma cell line. *J. Molec. Biol.*, **79**, 577.